# Studying the Hurdles of Insulin Prescription (SHIP^©^): development, scoring and initial validation of a new self-administered questionnaire

**DOI:** 10.1186/1477-7525-5-53

**Published:** 2007-08-29

**Authors:** Luc Martinez, Silla M Consoli, Louis Monnier, Dominique Simon, Olivier Wong, Bernard Yomtov, Béatrice Guéron, Khadra Benmedjahed, Isabelle Guillemin, Benoit Arnould

**Affiliations:** 1 French Society of General Medicine, Issy les Moulineaux, France; 2 Clinical Psychology and Psychiatric Department, Georges Pompidou European Hospital, University of Medicine Paris 5, Paris, France; 3 Endocrinology and Metabolisms Department, Lapeyronie Hospital, Montpellier, France; 4 Diabetology Department, Pitié Salpétrière Hospital, Paris, France; 5 Paris College of Family Physicians, Paris, France; 6 Diabetology Department, Henri Mondor Hospital, Créteil, France; 7 Medical and Scientific Division, Pfizer, Paris, France; 8 Mapi Values, Lyon, France

## Abstract

**Background:**

Although insulin therapy is well-accepted by symptomatic diabetic patients, it is still often delayed in less severe patients, in whom injectable insulin remains under-used. A better understanding of patients' perception of insulin would eventually help physicians to adopt the most appropriate dialogue when having to motivate patients to initiate or to intensify insulin injection.

**Methods:**

The 'Studying the Hurdles of Insulin Prescription' (SHIP) questionnaire was developed based on a list of concepts derived from three diabetic patients' focus groups, and was included into two cross-sectional studies with similar design: SHIP Oral study and SHIP Premix study. Diabetic patients treated with oral hypoglycaemic agents (OHA; n = 1,494) and patients already treated with insulin (n = 1,150) completed the questionnaire at baseline, 6- and 12 months. Psychometric properties were assessed: 1) structure analysis by Principal Component Analysis (PCA) with Varimax rotation, 2) internal consistency reliability (Cronbach's alpha), and 3) concurrent validity (Spearman correlation coefficients with the Fear of Self-Injecting (FSI) score of the Diabetes Fear of Injecting and Self-testing Questionnaire. Reluctance/motivation towards insulin was assessed. Scores' ability to predict patients' insulin injection reluctance/motivation and initiation/intensification was evaluated with the Area Under the Receiver Operating Characteristic (ROC) Curve (AUC).

**Results:**

PCA analysis confirmed the structure of the 14 items grouped into 3 dimensions: 'acceptance and motivation', 'fear and constraints', and 'restraints and barriers' towards insulin injection. Internal consistency reliability was excellent (Cronbach's alpha > 0.70); concurrent validity was good. The three scores were significantly predictive of patients' reluctance/motivation towards insulin injection initiation, as they were of patients' actual switch, except for the 'restraints and barriers' dimension. 'Acceptance and motivation' and 'fears and constraints' dimensions were also significantly predictive of patients' reluctance/motivation towards insulin intensification. By the end of the 12-month study, 179 of the initially OHA-treated patients had started insulin injections; 186 of the patients already treated with insulin had increased their injections.

**Conclusion:**

The SHIP questionnaire provides reliable and valid assessment of diabetic patients' attitude towards insulin and injections. The predictive power of scores for patients' reluctance/motivation and actual treatment decisions demonstrates encouraging potential for further application in clinical practice.

## Background

Diabetes constitutes a major healthcare problem worldwide. It is a highly prevalent disease, still increasing due to population aging and growth, together with rising obesity and physical inactivity [1,2]. Most patients have type 2 diabetes with poor control level, leading to increased morbidity and mortality rates caused by complications [3]. The economic burden of diabetes is highly significant, due to direct medical costs along with indirect costs related to a loss of productivity and the chronic aspect of the disease [4-6]. There is increasing evidence showing that improved blood glucose control decreases diabetic complications [7-9]. Specifically, it has been reported that improved blood glucose control with insulin or sulfonylurea therapy decreased the progression of microvascular complications in patients with type 2 diabetes [10].

Oral anti-diabetic therapy is the most commonly used treatment for type 2 diabetes, but its long-term efficacy is limited. Despite being well-accepted by symptomatic diabetes patients, insulin therapy is still often delayed in less severe patients, and is rarely used as an alternative treatment [6,10-13]. Given the benefits of intensive therapy combining oral anti-diabetic drugs plus insulin, this delay and low acceptance for insulin therapy is concerning. According to Brunton et al., both provider and patient fear is one contributing factor, with insulin therapy viewed as a "last resort" treatment option for severe disease [14]. Patients delay insulin therapy because of the lack of obvious symptoms at the beginning of the disease, and because of physicians' fears about reducing the quality of life of patients when starting insulin [15]. This may in turn have important negative impacts on patients' well-being [16]. Yet, a two-year prospective descriptive study showed that insulin therapy initiation in relatively asymptomatic type 2 diabetes patients who were treated with diet and/or hypoglycaemic agents resulted in improved glycaemia control, without major adverse influences on patients' quality of life [15]. A recent randomised controlled trial reported that patients who were offered inhaled insulin opted more frequently for a treatment that included insulin than patients in the control group who were offered standard treatments [17].

Questionnaires exist that assess perceptions of diabetes patients regarding insulin therapy. Amongst them, the Insulin Delivery System Rating Questionnaire (IDSRQ) and the Insulin-Therapy-Related Quality of Life questionnaire (ITR-QoL) are specifically designed to measure patients' satisfaction with insulin delivery systems and/or their preference [18,19]; others, such as the Diabetes Treatment Satisfaction Questionnaire (DTSQ), the Insulin Treatment Satisfaction Questionnaire (ITSQ) and the triad of measures (i.e. Satisfaction Measure, Symptom Measure and Productivity Measure) proposed by Brod and colleagues aim at assessing patients' satisfaction with insulin treatment regimen [20-22]. Cappelleri et al. highlighted the contribution of treatment convenience and ease of use and social comfort as significant factors in diabetes patients' satisfaction [23]; recent studies from Brod and al. identified several factors (e.g. age, co-morbidity, treatment efficacy, weight gain) as contributing to patients' satisfaction [24], and suggested that satisfaction is not a static concept [25]. But so far, there is no questionnaire allowing the elicitation of patients' expectation and attitude towards insulin therapy, as well as predicting patients' intentions regarding insulin therapy. A better understanding of the reasons why patients so poorly accept insulin injection therapy would allow physicians to adopt the most appropriate behaviour and dialogue when having to motivate patients to initiate subcutaneous insulin injections or to intensify the number of insulin injections.

In this paper, we present the development and psychometric validation of a new self-administered instrument, the Studying the Hurdles of Insulin Prescription (SHIP^©^) questionnaire. The questionnaire aims at exploring motivation, fears, and barriers towards insulin injection therapy (type 2 diabetes patients treated with oral hypoglycaemic agents) or towards intensifying injections (type 1 or type 2 diabetic patients already treated with insulin injection). The ability of the questionnaire to predict patients' intentions regarding initiating or intensifying their insulin treatment, either as an injection or an inhalation, was evaluated.

## Methods

### Development of the SHIP^© ^questionnaire

**Figure 1:**
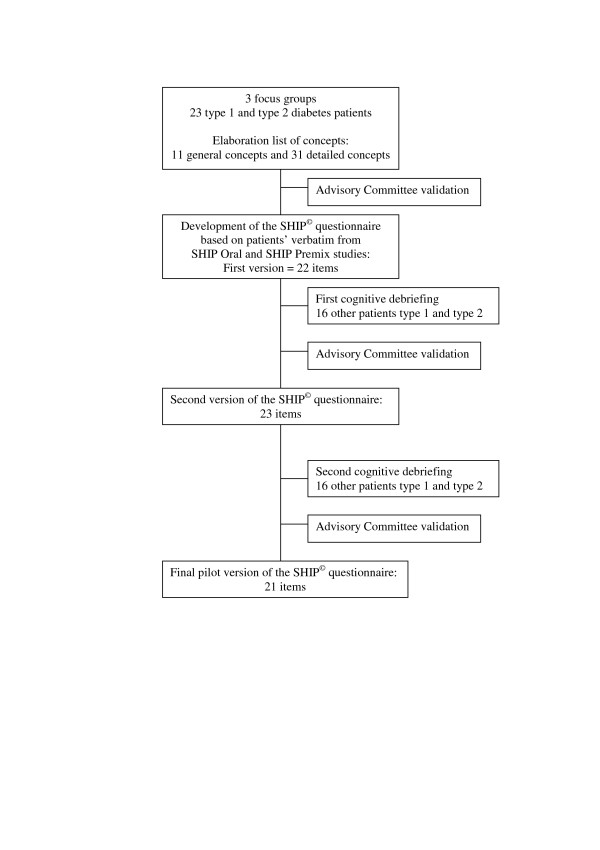
Overview of the qualitative phase of SHIP^© ^questionnaire development.

This qualitative phase led to the item generation and development of the pilot questionnaire. The overview of the phase is represented in Figure 1. First, three focus groups of type 1 and type 2 diabetes patients were used to capture fears, constraints and benefits regarding insulin therapy, insulin injections and insulin regimen step-up [26]. A list of detailed concepts was established from patients' own words, from which a test questionnaire was developed and validated by the Advisory Committee (AC) consisting of three diabetes specialists/endocrinologists, one psychiatrist and two general practitioners. The wording of the questionnaire was adapted to assess either the attitude of patients currently treated with oral hypoglycaemic agents (OHA) regarding a switch to insulin therapy, or the attitude of patients already treated with premix insulin regarding a step-up in the number of insulin injection. The item content of the SHIP^© ^questionnaire is presented in Table 1. Its content validity was assessed twice, using patient cognitive debriefing among type 1 and type 2 diabetes patients [27]. The resulting pilot questionnaire was validated by the AC.

**Table 1:** Item contents of the SHIP^© ^questionnaire and Pearson correlation coefficients between items and dimensions performed with cross-sectional populations at baseline for SHIP Oral and SHIP Premix studies. Correlations > 0.40 are in bold

**Item contents**	**SHIP Oral Study (n = 1,478^a^)**				**SHIP Premix Study (n = 1,130^b^)**			
		
	**Missing Data N (%)**	**Acceptance Motivation**	**Fears Constraints**	**Restraints Barriers**	**Missing Data N (%)**	**Acceptance Motivation**	**Fears Constraints**	**Restraints Barriers**
Willingness because of more balanced diabetes	12 (0.80)	**0.64**	0.20	0.20	21 (1.83)	**0.63**	0.23	0.13
Happy because of improvement in quality of living	11 (0.74)	**0.63**	0.23	0.15	20 (1.74)	**0.62**	0.23	0.06
Favourable because of easiness to use of treatment	14 (0.94)	**0.64**	0.31	0.27	20 (1.74)	**0.68**	0.32	0.23
Advantage because of a less restrictive diet	NA^c^	**0.57**	0.12	0.11	20 (1.74)	**0.50**	0.08	-0.02
Confidence in physicians	23 (1.54)	**0.55**	0.11	0.24	20 (1.74)	**0.56**	0.17	0.18
Feeling restricted because of self-surveillance	18 (1.20)	0.23	**0.63**	0.32	13 (1.13)	0.28	**0.67**	0.35
Constraint because of dependency, liberty loss	17 (1.14)	0.21	**0.63**	0.26	22 (1.91)	0.21	**0.62**	0.30
Upset diabetes is getting worse	15 (1.00)	0.04	**0.41**	0.21	22 (1.91)	0.07	**0.47**	0.25
Fear of having more hypoglycaemia crises	15 (1.00)	0.11	**0.45**	0.37	22 (1.91)	0.08	**0.48**	0.23
Fear that treatment gets more complicated	15 (1.00)	0.35	**0.58**	0.41	18 (1.57)	0.38	**0.60**	0.42
Bothered by being seen while injecting insulin	21 (1.41)	0.24	0.35	**0.71**	17 (1.48)	0.10	0.30	**0.61**
Fear that people notice I'm diabetic	16 (1.07)	0.17	0.34	**0.57**	19 (1.65)	0.08	0.34	**0.57**
Bothered by skin being marked at injection site	12 (0.80)	0.13	0.29	**0.56**	14 (1.22)	0.12	0.28	**0.47**
Stressed because injections can be painful	14 (0.94)	0.24	0.39	**0.53**	20 (1.74)	0.14	0.35	**0.48**

^a ^1,478 = 1,487-9, due to missing data

^b ^1,130 = 1,141-11, due to missing data

^c ^NA, not assessed

### Study design and patient populations

The pilot SHIP^© ^questionnaire was administered in two French multicentre cross-sectional studies conducted in parallel, with identical design but different populations of patients.

#### SHIP Oral Study

Four hundred and twelve random general practitioners included about 1,500 ambulatory type 2 diabetes patients treated with at least two OHAs. Pregnant women and type 1 diabetes patients were excluded from the study. Type 2 diabetes patients treated with no or only one OHA, and those whose regimen added insulin were also excluded.

#### SHIP Premix study

Three hundred random diabetologists and endocrinologists included about 1,500 type 1 and type 2 diabetes patients treated with two daily injections of Premix insulin. Type 2 patients treated with OHAs only or with insulin other than two premix injections were excluded from the study.

For both studies, patients self-completed the SHIP^© ^questionnaire at their first inclusion visit, at their physicians' practice. In order to assess the proportion of patients presenting symptoms of fear of injections, they were also asked to complete the Diabetes Fear of Injecting and Self-testing Questionnaire (D-FISQ) at baseline [28]. In parallel, patients had to fill out a record card for blood glucose control and hypoglycaemic treatment at the 6- and 12-month follow-up visits. At each of the visits, patients who did indeed start insulin injection treatment and those who intensified the number of injections were counted.

Each of the two populations was randomly divided into two subgroups based on a 2:1 ratio. One subgroup (two thirds of the total population) was used for the finalisation step (to shorten the pilot questionnaires and to define the process of scoring) and will be referred to as the 'finalisation dataset'; the second subgroup (one third of the total population) was used for the validation step, and will be referred to as the 'validation dataset'. Psychometric properties were defined with the corresponding cross-sectional populations of the two studies.

### Statistical analysis

Principal Components Analysis (PCA) with Varimax Rotation was used to assess the SHIP^© ^questionnaire structure in each study. Floor and ceiling effects were examined, and psychometric properties were further assessed. This included a Multitrait Analysis (MA) that evaluated the final structure of the questionnaire by describing item convergent validity and item discriminant validity [29]. Convergent validity was confirmed when correlations between each item and its own scale were ≥ 0.40 [30]. Internal consistency reliability was determined using Cronbach's alpha coefficient [31], values greater than 0.70 indicating a high level of internal consistency [32]. Concurrent validity was evaluated between the Fear of Self-Injecting (FSI) score of the D-FISQ and the SHIP^© ^questionnaire by calculating Spearman correlation coefficients [28]. Correlation coefficients ranging between 0.4 and 0.7 were considered as reflecting similar but no redundant concepts [33]. The discriminative power of the questionnaire was established by comparing groups of patients based on age, gender, type of diabetes, time since patients' diabetes had been diagnosed, HbA1c dosage, and the number of times physicians have talked about insulin therapy with their patients.

Descriptive analyses were completed by comparative tests (Mann-Whitney Wilcoxon and Kruskal-Wallis tests) for qualitative parameters, and by Spearman coefficient correlations for quantitative parameters.

The ability of the SHIP^© ^questionnaire to predict actual change in patients' treatment regimen (initiation or intensification of insulin injections), as well as patients' intention (reluctance-motivation) to initiate or to increase insulin injections or inhaled insulin at the end of their inclusion visit, at 6 months and at 12 months was predicted by performing three univariate logistic regressions with each of the scores as a covariate. The predictive validity was determined by measuring the Area Under the Receiver Operating Characteristic (ROC) Curve (AUC) [34]. AUC was considered acceptable when higher than 0.70. First, univariate logistic regressions were realised in the 'finalisation dataset' in order to define the AUC for each of the items. Then, univariate logistic regressions were performed in the 'validation dataset', on the final questionnaire, in order to assess the predictive ability of each dimension. For the intention regarding injected or inhaled insulin items, patient responses were divided into two groups: one containing the two negative modalities ("I would refuse" and "I would be rather reluctant", respectively), and the other containing the two positive modalities ("I would be quite motivated" and "I would be highly motivated").

Statistical analyses were performed using Statistical Analysis Software version 8.02 for Windows. The significance level of the tests was fixed at 0.05.

## Results

### Development of the SHIP^© ^questionnaire

Eleven global concepts covering insulin therapy (benefits; symbolic meaning; fears; constraints; products' characteristics), insulin injections (symbolic meaning; fears; constraints; advantages), and intensification of insulin injections (symbolic meaning; fears), divided into 31 detailed concepts were identified from the focus groups. Based on these concepts, 22 items were designed, comprising 20 items related to the insulin regimen initiation/intensification and two items related to inhaled insulin. Following the first cognitive debriefing, one item was added and the response choices were significantly modified in order to facilitate the understanding of the questionnaire by patients. Following the second cognitive debriefing, two items were deleted. Finally, the SHIP^© ^questionnaire was constituted of 21 items: 18 items evaluated patient attitude towards initiation (SHIP Oral study) or intensification (SHIP Premix study) of insulin injections; one item asked patients to select 5 items they found the most important out of the 18 previously answered; one item assessed patient intentions regarding either initiation or intensification of insulin injections; one item covered patient intentions regarding inhaled insulin if available. The structure of the questionnaire and item content were identical in both studies. However, the items' formulation slightly differed to fit patients' actual treatment; precisely, the word "initiation" was used in the SHIP Oral study versus "intensification" in the SHIP Premix study.

### Study population

Among the 1,494 type 2 patients (SHIP Oral study) and the 1,150 type 1 and type 2 patients (SHIP Premix study) recruited, respectively 1,487 (99.5%) and 1,141 (99.2%) questionnaires were assessable (i.e. completed with less than 50% missing data).

The number and percentages of missing data according to the items of the questionnaire are summarised in Table 1. Percentages of missing data ranged from 0.60 to 1.54 for SHIP Oral, and from 1.13% to 3.91% for SHIP Premix.

Concerning patients included in SHIP Oral and SHIP Premix studies, the overall mean ages were 63.9 years and 62.0 years, respectively; the proportions of male patients were 57% and 52%; on average, patients had been diagnosed 10 and 16 years ago; mean body mass index (BMI) was 30 kg/m^2 ^and 29 kg/m^2^. The majority of patients (75.2%) recruited for the SHIP Premix study had type 2 diabetes and had been treated with insulin for five years on average. Thirty six percent of patients from the SHIP Oral and 57% from the SHIP Premix studies had complications associated with their diabetes. Mean HbA1c at inclusion was respectively 7.4% and 7.9%.

### Patients' intentions and actual changes in treatment regimen

As observed from the responses of patients treated with OHA to the questionnaire's item at the inclusion visit, the majority of patients (70%) would be "rather reluctant" to "reluctant" to start insulin injection; only 1.6% indeed started it at the end of their inclusion visit. A large majority of patients (83%) would have been "quite motivated" to "highly motivated" to initiate an insulin treatment if inhaled. Ninety-six percent of patients who were favourable to injected insulin were also favourable to inhaled insulin. Concerning the SHIP Premix study, 63% of patients were "quite motivated" to "highly motivated" to increase the number of insulin injections. Again, a large majority of patients (81%) would have been motivated to increase their insulin therapy treatment if inhaled insulin was available. At the end of their inclusion visit, 40% of these patients had a change in their insulin treatment, but only 13% corresponded to an increased number of injections.

Of all patients treated with OHA (n = 1,487), only 24 did indeed initiate insulin injections at baseline. At the 6-month follow-up visit, 98 additional patients joined the group of patients already injecting insulin, and 57 additional patients initiated insulin injections at the 12-month follow-up visit, thus resulting in a total of 179 patients (12% of the total cross-sectional population) having started insulin injections by the end of the 12-month study. 1,043 patients were reluctant to initiate an insulin treatment by injection, and 247 to initiate inhaled insulin therapy. Of patients already treated by insulin injection (n = 1,141), 143 increased their number of injections at the end of the inclusion visit; at the 6-month visit, 25 decreased the number of injections, while 70 additional patients increased them, resulting in 188 patients having increased the number of injections. Of these 188 patients, 67 patients increased the number of injections and 69 decreased them at the 12-month visit, thus resulting in a total of 186 patients (corresponding to 16% of the cross-sectional population) with an increased number of insulin injections by the end of the 12-month study. At this point, 427 patients were reluctant to increase the number of insulin injections and 198 were reluctant to increase their treatment even with inhaled insulin.

### Finalisation of the SHIP^© ^questionnaire: item reduction

The finalisation of the questionnaire was performed on the 'finalisation dataset' (n = 992 for the SHIP Oral study and n = 761 for the SHIP Premix study).

Based on a first PCA analysis with Varimax Rotation and MA that were performed separately on the questionnaire filled out at baseline, four items were eliminated as they displayed poor discriminant or convergent validity, had low predictive value, or were not adapted to patients under insulin therapy without OHA. Three factors comprising 14 items were identified from the PCA analysis: the first factor corresponded to items about patients' acceptance and motivation for insulin; the second factor contained items about patients' fears and constraints regarding insulin therapy; the third factor contained items about restraints and barriers patients perceived with insulin therapy.

### Validation of the SHIP^© ^questionnaire: scoring

In order to validate the new structure of the questionnaire, second PCA and MA analyses were performed with the 'validation dataset'. The three factors and 14 items were confirmed, dealing with either insulin injection initiation (SHIP Oral study) or insulin injection intensification (SHIP Premix study). The three dimensions of the questionnaire were thus named respectively 1) 'acceptance and motivation', 2) 'fears and constraints' and 3) 'restraints and barriers' towards injection (Table 1).

Scoring of the SHIP^© ^questionnaire was based on the standardised sum of the items within one dimension, giving a range from 0 (lowest level for the dimension assessed) to 100 (highest level). The distribution of SHIP^© ^questionnaire scores from both SHIP Oral and SHIP Premix studies are shown in Table 2.

**Table 2:** Score distribution (± standard deviation) of each of the dimensions obtained from cross-sectional populations at baseline

Dimensions	SHIP Oral study (N = 1,487)	SHIP Premix study (N = 1,141)
'Acceptance and motivation'	47.1 ± 25.3	62.5 ± 26.4
'Fears and constraints'	69.3 ± 24.9	58.2 ± 28.2
'Restraints and barriers'	35.5 ± 28.6	23.5 ± 25.4

### Instrument psychometric properties

#### Construct validity

The final structure of the SHIP^© ^questionnaire was tested by performing PCA analysis followed by MA, which validated the division of the questionnaire into three dimensions. Item-dimension correlations were determined (Table 1). Correlations of each of the items with its own dimension were all higher than the threshold value of ≥ 0.40. All items also satisfied the divergent validity criteria.

#### Internal consistency reliability

Table 3 summarises the data of the SHIP^© ^questionnaire completed by patients of the two cross-sectional studies at baseline. Cronbach's alpha coefficients were good and similar, ranging from 0.77 to 0.82 for the SHIP Oral study, and 0.74 to 0.81 for the SHIP Premix study.

**Table 3:** Internal consistency reliability of the SHIP^© ^questionnaire dimensions at baseline, in SHIP Oral (N = 1,478^a^) and SHIP Premix (N = 1,130^b^) studies, as measured by Cronbach's alpha

Dimensions	SHIP Oral study	SHIP Premix study
'Acceptance and motivation'	0.82	0.81
'Fears and constraints'	0.77	0.79
'Restraints and barriers'	0.78	0.74

^a ^N = 1,487-9 due to missing data

^b ^N = 1,141-11 due to missing data

#### Concurrent validity

The correlation between the FSI score and the 'acceptance and motivation' dimension score of SHIP Premix was the weakest (Spearman coefficient = -0.18); correlation was higher and similar between FSI score and 'fears and constraints' and 'restraints and barriers' dimension scores in both studies (Spearman coefficients = 0.41 and 0.42, respectively).

#### Predictive power of the SHIP^© ^questionnaire scores

**Figure 2:**
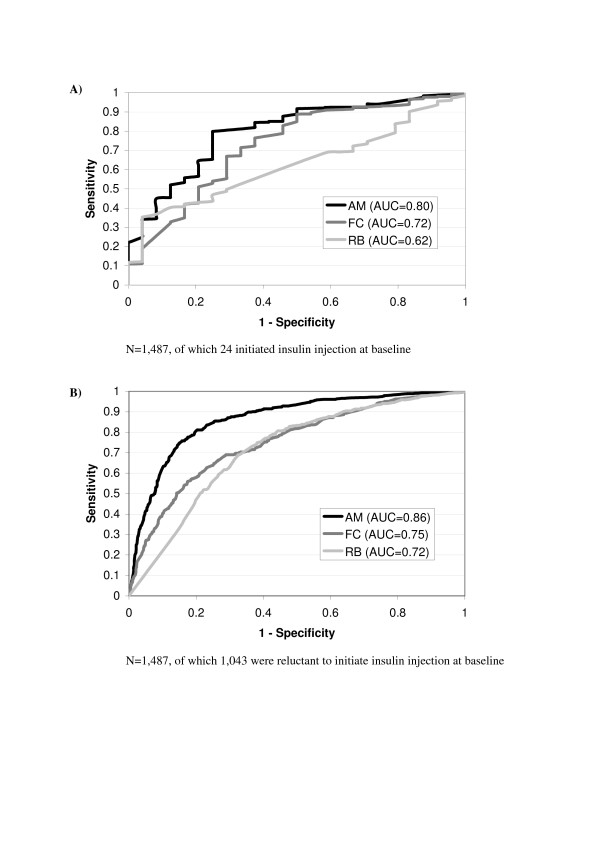
ROC Curve of the three scores about patients' attitude towards initiating insulin injection (A) and their intentions regarding insulin therapy (B) at baseline; ability of the questionnaire to predict type 2 diabetes patients' attitude and intentions regarding initiating insulin injection are deduced from the Area Under the Curve (AUC). AM, 'acceptance and motivation'; FC, 'fears and constraints'; RB, 'restraints and barriers'

Figures 2A and 2B represent the ROC curves drawn to predict patients' intentions regarding insulin initiation and actual change in treatment regimen for each dimension score at baseline. AUCs are summarised in Table 4. In the SHIP Oral study, AUC values of the 'acceptance and motivation' dimension and to a lesser extent, the 'fears and constraints' dimension, were the highest for predicting clinicians' decisions about insulin injection initiation and patients' intentions regarding insulin injection treatment at baseline. These values were lower than 0.70 at 6- and 12-month follow-up visits. Value of the 'restraints and barriers' dimension for predicting clinicians' decisions about insulin injection initiation was low at baseline, and slightly higher for predicting patients' reluctance to initiate insulin injection (0.62 and 0.72, respectively). Values to predict patients' reluctance regarding initiation of inhaled insulin were lower than 0.70 for all three dimensions.

**Figure 3:**
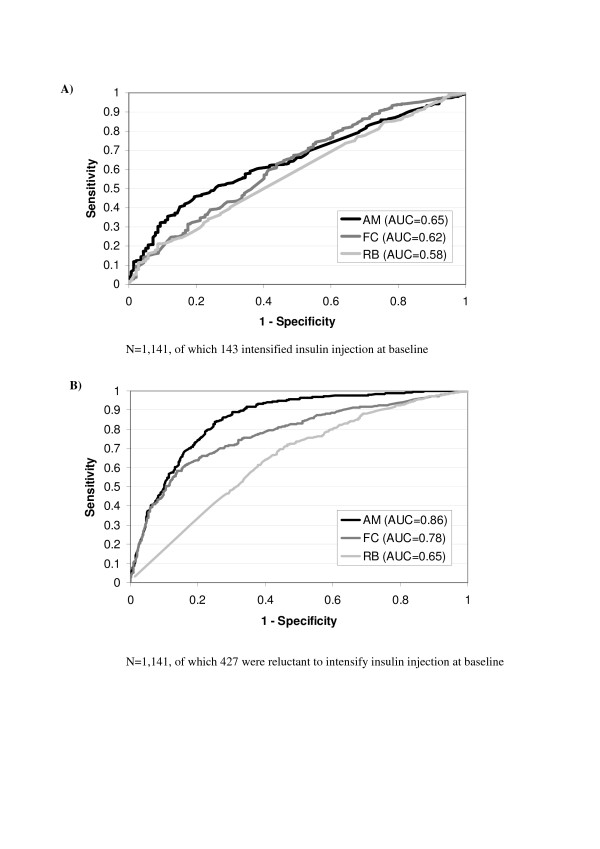
ROC Curve of the three scores about patients' attitude towards intensifying insulin injection (A) and their intentions regarding insulin therapy (B) at baseline; ability of the questionnaire to predict patients' attitude and intentions regarding intensifying insulin injection are deduced from the Area Under the Curve (AUC). AM, 'acceptance and motivation'; FC, 'fears and constraints'; RB, 'restraints and barriers'

In the SHIP Premix study, AUCs of the three dimensions were all lower than 0.70, ranging from 0.58 to 0.65, for assessing patients' switch to injection intensification at baseline and the two follow-up visits (Figures 3A and 3B and Table 4). Only the 'acceptance and motivation' and 'fears and constraints' dimensions showed fair ability to predict patients' intentions regarding insulin intensification, and the 'acceptance and motivation' dimension to predict attitude towards intensification if inhaled insulin was available.

**Table 4:** Predictive power measured by the AUC of each dimension scores of the questionnaire in the SHIP Oral and SHIP Premix studies for patients' attitude and actual choice and behavioural intentions regarding insulin therapy

	SHIP Oral study/SHIP Premix study				
	
Dimensions	Clinician actual treatment decision regarding insulin initiation/Intensification			Patients' intention regarding	
				insulin injection initiation/intensification	inhaled insulin initiation/intensification
	
	Inclusion	6 months	12 months	Inclusion	Inclusion
'Acceptance and motivation'	0.80/0.65	0.65/0.64	0.59/0.64	0.86/0.86	0.65/0.72
'Constraints and fears'	0.72/0.62	0.58/0.61	0.56/0.60	0.75/0.78	0.60/0.68
'Restraints and barriers'	0.62/0.58	0.59/0.60	0.58/0.61	0.72/0.65	0.57/0.63

#### Scores of the SHIP^© ^questionnaire according to specific subgroups

In the SHIP Oral study, the 'acceptance and motivation' score was significantly higher in men than in women (scores = 49 and 45, respectively; p = 0.013) and in younger people (p = 0.006), with scores decreasing in older subjects. On the contrary, 'fears and constraints' towards insulin therapy scores were significantly higher in women and increased significantly in older subjects. 'Restraints and barriers' scores were significantly higher in women and decreased in older patients. The 'acceptance and motivation' score was significantly higher in patients recently diagnosed, and progressively decreased as the time since diagnosis increased. 'Fears and constraints' and 'restraints and barriers' scores did not show significant differences. When observed according to HbA1c dosage, none of the 3 dimension scores showed significant differences. 'Acceptance and motivation' scores were directly correlated to the answer given by patients about their intention regarding initiating insulin injections: as patients motivation increased, so did scores (scores = 31 and 43 for patients who "would refuse" and who "would be rather reluctant", respectively; scores = 67 to 75 for patients "quite motivated" to "highly motivated"). On the other hand, 'fears and constraints' and 'restraints and barriers' scores significantly decreased as patients' motivation increased (scores = 75 and 46 for patients who answered they "would refuse" to start injections; scores = 50 and 19 for "highly motivated" patients; p < 0.0001).

In the SHIP Premix study, 'acceptance and motivation' scores were significantly different according to patients' age, with the lowest score displayed by the youngest and oldest patients (i. e. 59 for < 40 years-old patients and 60 for > 70 years-old; p = 0.023). 'Restraints and barriers' scores were significantly (p < 0.0001) different between age groups, and decreased with patients getting older. No significant difference was observed for the 'fears and constraints' scores. When compared according to the type of diabetes, type 1 patients had a lower 'acceptance and motivation' score than type 2 diabetes patients (scores of 59 versus 64, respectively; p = 0.01). On the other hand, 'restraints and barriers' scores were higher for type 1 diabetes patients than for type 2 (p < 0.0001; scores of 29 versus 22). 'Fears and constraints' and 'restraints and barriers' scores significantly (p < 0.05) increased for patients who talked about insulin therapy with their physicians ('fears and constraints' and 'restraints and barriers' to injection scores = 57 and 21, respectively, for patients whose physicians never talked to them about insulin therapy; scores = 61 and 27, respectively for patients whose physicians talked to them several times). 'Acceptance and motivation' scores were not significantly different (p = 0.0916). None of the three scores showed significant difference when compared to the groups based on HbA1 dosage. As in the SHIP Oral study, when compared to patients' responses to the item on intention regarding injection intensification, the 'acceptance and motivation' score was directly correlated to patients' level of motivation: the higher the patients' motivation, the higher the scores (scores = 33 and 48 for patients who "would refuse" and who "would be rather reluctant", respectively; scores = 72 to 81 for patients "quite motivated" to "highly motivated"; p < 0.0001). On the contrary, scores of 'fears and constraints' and 'restraints and barriers' to insulin therapy were inversely correlated: the lower the patients' motivation, the higher the score (scores = 71 and 34, respectively, for patients who answered they "would refuse" to start injections; scores = 42 and 18 for "highly motivated" patients; p < 0.0001).

## Discussion

The SHIP^© ^questionnaire was developed in order to assess and predict patients' attitude, intentions and actual change in treatment regimen regarding insulin injection initiation/intensifications by identifying and evaluating factors defining patients' perception, both positive and negative, of insulin therapy and insulin injections in particular.

The questionnaire is concise, containing 14 items grouped into 3 dimensions that cover 'acceptance and motivation' (5 items), 'fears and constraints' (5 items), and 'restraints and barriers' (4 items). One item asked patients to indicate the 5 items they found the most important out of the 14 previously answered items. Two additional items evaluated patients' intentions regarding insulin initiation or intensification, either injected or inhaled. The development of the questionnaire followed a standardised and rigorous methodology [26,27], which ensured its good content and construct validity and its good acceptability. Item convergent and discriminant validity were satisfactory for all items, indicating that each of them assessed the concept of its own dimension without redundancy between dimensions. Internal consistency reliability of the dimensions proved to be excellent. The questionnaire showed good correlations but no redundancy between the concepts measured and the fear of self-injecting factor of D-FISQ [28]. Lastly, the SHIP^© ^questionnaire showed ability to predict patients' intentions regarding insulin therapy as well as their actual change in treatment regimen, in the SHIP Oral study and to a lesser extent, in the SHIP Premix study.

Overall, the more recently diagnosed the diabetes, the more motivated the patients are to initiate insulin injections. Among them, men were more motivated than women, consistent with the higher level of restraints, fears and constraints that women referred to when facing such treatment. This suggested that women are probably more concerned than men about the image they give of themselves to others. Patient motivation and acceptance of the treatment were consistent with their attitude towards insulin injections, i.e. motivated patients presenting low restraints and fears had the highest positive attitude towards such treatment.

As expected, patients who were already receiving insulin injections were in large majority less reluctant to increase the number of injections than patients who were receiving treatment orally (70% versus 37%). This observation was directly reflected by the higher proportion of patients already treated by insulin injections who did indeed undergo insulin injection intensification compared to patients orally treated who did not initiate insulin injection, regardless the time of the study (13% versus 2% at baseline; 16% versus 8% after 6 months, 16% versus 12% after 12 months). These orally treated patients were highly concerned about the fears and constraints related to insulin injections, with 13% of them reporting they would be worried that their diabetes would get worse, that they would have more hypoglycaemic incidents, that they would feel more dependent, or that their treatment would get more complicated. Although to a lesser extent, same reasons were reported by patients who would later agree to increase the number of insulin injections. Similarly, twice more patients already treated with insulin injection would agree to increase their treatment if inhaled insulin was available, compared to patients treated with OHA (37% versus 17%, respectively).

Interestingly, in the SHIP Oral study, the 3 dimensions of the questionnaire were predictive of patients' intentions regarding insulin: the more motivated the patients and the lower their fears and the barriers, the less reluctant the patients were to initiate insulin injection. In the same way, at baseline, the level of motivation and fears predicted the intentions of patients already treated with insulin injections regarding an increased number of injections. In the SHIP Premix study, the questionnaire was not predictive of patients who actually underwent an increased number of injections. Restraints and barriers were good predictive criteria of patients' intentions regarding initiation of insulin injections but in contrast, could not predict their intentions regarding treatment intensification. One could propose that at the inclusion visit, patients who were already convinced or were about to be convinced, were highly motivated to start/intensify insulin injection; on the contrary, patients who were the most worried about insulin treatment were not motivated, and indeed did not start/intensify insulin injections, and required more interactions with their physicians. That is, in order for patients to change to and fully accept a new treatment, the most important concern is for them to be convinced. At 6- and 12-month visits, patients have had time to think about insulin injection treatment and be "mentally prepared" for it. Thus, patients' responses at baseline no longer reflect their actual state of mind 6 or 12 months later. Furthermore, patients' medical status may have evolved, resulting in a physician's decision that differs from patients' intentions at their first visit. The 'restraints and barriers' dimension was not predictive of the treatment eventually administered, even at baseline. This further confirmed that in order to trigger patient decision, physicians should particularly insist on the positive aspects of an efficient treatment, as patients essentially need to be convinced. The slightly higher predictive ability of the questionnaire in the SHIP Premix study compared to that in the SHIP Oral study could be explained by the fact that patients who receive insulin injections are already familiar with this mode of administration; in contrast, patients in the SHIP Oral study are insulino-naïve, and therefore lack information about the advantages and inconveniencies of such treatment.

Altogether, these observations on patient attitude, intentions, and behaviour towards insulin treatment follow and share features of the Theory of Planned Behaviour model, a theory from social psychology field [35-37]. According to this theory, behavioural intention is a good proximal measure of actual behaviour, and in order to predict a person's behavioural intention, information on 'attitude' (i.e. whether a person is in favour of doing 'it'), 'subjective norms' (i.e. how much a person feels social pressure to do 'it') and 'perceived behavioural control' (i.e. whether the person feels in control of the action) is required. By allowing this information to be assessed, the SHIP^© ^questionnaire provides good basis for further identifying how patients' management at an individual or collective level could help to influence their behaviour, as well as for facilitating clinicians' decision to switch a patient's treatment to insulin or to increase insulin doses whenever required. The impact of its use to support improved communication and care needs to be evaluated in a specific study.

Concerning inhaled insulin, the SHIP^© ^questionnaire did not demonstrate convincing findings in its ability to help predict patients' intentions in any of the studies. However, as already observed in previous work [17], it is interesting to note that patients, regardless of their type of diabetes and insulin therapy, would be very much in favour of inhaled insulin if this were available.

## Conclusion

The design of this present study (i.e. large population samples and long time period), the rigorous methodology used to develop the questionnaire, the characteristics of its structure and clinical content, and the properties of the instrument make the SHIP^© ^questionnaire a good candidate for further validation of its use in clinical practice. Larger studies with specific population settings and real-life studies will be useful to validate and confirm its place in everyday and clinical practice [38].

The SHIP^© ^questionnaire is a reliable and valid instrument that is promising for assessing the intentions of type 1 and type 2 diabetes patients regarding insulin therapy, and the reasons for their behaviour. The questionnaire demonstrates ability to predict actual change in treatment regimen in the short term, and confirms the importance of patient-physician communication in treatment decision in diabetes. The SHIP^© ^questionnaire would thus be a helpful tool for physicians to interact and communicate with their patients.

## Copyrights

The SHIP^© ^questionnaire is protected by copyright with all rights reserved by Pfizer. Do not use without permission. For information on, or permission to use the USP^©^, please contact the Mapi Research Trust, 27 rue de la Villette 69003 Lyon, FRANCE. Tel: +33 (0) 472 13 65 75 - E-mail: trust@mapi.fr - website: www.mapi-research.fr.
